# Decreasing *Wapl* dosage partially corrects embryonic growth and brain transcriptome phenotypes in *Nipbl^+/−^* embryos

**DOI:** 10.1126/sciadv.add4136

**Published:** 2022-11-30

**Authors:** Connor M. Kean, Christopher J. Tracy, Apratim Mitra, Beenish Rahat, Matthew T. Van Winkle, Claudia M. Gebert, Jacob A. Noeker, Anne L. Calof, Arthur D. Lander, Judith A. Kassis, Karl Pfeifer

**Affiliations:** ^1^ Division of Intramural Research, Eunice Kennedy Shriver National Institute of Child Health and Human Development, National Institutes of Health, Bethesda, MD, USA.; ^2^ Department of Anatomy and Neurobiology, University of California School of Medicine, Irvine, CA, USA.; ^3^ Department of Developmental and Cell Biology, University of California, Irvine, Irvine, CA, USA.

## Abstract

Cohesin rings interact with DNA and modulate the expression of thousands of genes. NIPBL loads cohesin onto chromosomes, and WAPL takes it off. Haploinsufficiency for *NIPBL* causes a developmental disorder, Cornelia de Lange syndrome (CdLS), that is modeled by *Nipbl^+/−^* mice. Mutations in *WAPL* have not been shown to cause disease or gene expression changes in mammals. Here, we show dysregulation of >1000 genes in *Wapl*^Δ*/+*^ embryonic mouse brain. The patterns of dysregulation are highly similar in *Wapl* and *Nipbl* heterozygotes, suggesting that *Wapl* mutations may also cause human disease. Since WAPL and NIPBL have opposite effects on cohesin’s association with DNA, we asked whether decreasing *Wapl* dosage could correct phenotypes seen in *Nipbl^+/−^* mice. Gene expression and embryonic growth are partially corrected, but perinatal lethality is not. Our data are consistent with the view that cohesin dynamics play a key role in regulating gene expression.

## Teaser

Investigation of transcription in the brains of CdLS mouse models demonstrates the importance of *Nipbl*:*Wapl* balance.

## INTRODUCTION

The cohesin complex consists of the subunits SMC1, SMC3, RAD21, and Stromalin, which form a ring-like structure that encircles DNA (*1*). Cohesin’s interactions with DNA are dynamic (*2*–*4*). Cohesin is loaded onto chromosomes by the kollerin complex, which consists of Nipped-B–like (NIPBL) and MAU2 (*5*). Once loaded, cohesin can translocate along the chromosome (*4*, *6*, *7*) or be removed by the cohesin-releasing factors PDS5 and WAPL (*8*, *9*). Cohesin, stimulated by NIPBL, acts as an adenosine triphosphate (ATP)–dependent molecular motor extruding DNA and folding the genome into topologically associated domains (TADs) (*10*–*13*). Removing either PDS5 or WAPL stabilizes cohesin binding to chromatin and can alter TAD structure in different ways (*4*, *14*–*16*).

The cohesin complex is required for sister chromatid cohesion and ensures accurate chromosome segregation upon cell division (*17*, *18*). Thus, severe disruption of cohesin function results in aneuploidy and cell death. However, studies in *Drosophila*, zebrafish, mouse, and human reveal that reduced expression of cohesin subunits or of NIPBL alters gene expression and development without evident defects on sister chromatid cohesion and chromosome segregation (*19*–*23*). For example, Cornelia de Lange syndrome (CdLS) is caused by heterozygous loss-of-function mutations in *NIPBL* (*24*, *25*). CdLS patients display severe developmental defects that vary from patient to patient but always include neurodevelopmental delay and some degree of intellectual disability (*26*, *27*). Mutations in other proteins that alter cohesin function cause similar defects; the developmental syndromes caused by these mutations are collectively known as cohesinopathies [reviewed in (*1*, *28*, *29*)].

*Nipbl^+/−^* mice effectively phenocopy most key features of CdLS (*21*, *30*). Late-stage embryos are always smaller than wild-type littermates and display a range of developmental defects and organ abnormalities that occur with variable degrees of penetrance and severity (*23*). In an isogenic C57BL/6J background (as used in this study), *Nipbl* heterozygotes die perinatally. Even on an outbred background, survival is limited to about 20% of animals (*14*, *23*). As noted in CdLS patients, cell division in *Nipbl^+/−^* mice appears normal (*14*). Instead, mutant phenotypes are associated with changes in gene expression that are typically modest (<2-fold) but occur across hundreds of genes in every tissue tested (*14*).

Mutations in *NIPBL* account for most CdLS cases, and no cases of CdLS have yet been attributed to mutations in *WAPL* (*31*). However, compiled data from healthy individuals reveal a dearth of predicted loss-of-function mutations in *WAPL* coding sequences, suggesting that *WAPL* is haploinsufficient. In addition, a single de novo, heterozygous, missense mutation in *WAPL* was identified in a patient presenting with neurodevelopmental defects (*32*). Together, these findings suggest that *WAPL* heterozygosity, like *NIPBL* heterozygosity, might cause disease.

NIPBL loads cohesin on chromosomes, and WAPL removes it. Therefore, we wondered whether decreasing the dose of *Wapl* could correct phenotypes in *Nipbl^+/−^* mice. Previous studies in *Drosophila* had shown that decreasing the dosage of *Nipped-B* (the *Drosophila* homolog of *NIPBL*) could correct a developmental phenotype caused by a dominant-negative *Wapl* allele (*33*). Similarly, reducing *WAPL* function in human cell lines permitted the survival of cell lines lacking *NIPBL* and *MAU2* (*4*). Here, we generate and characterize novel mouse *Wapl* alleles. We examined the transcriptomes of *Wapl* heterozygotes and show that, like *Nipbl^+/−^* mice, the brains of *Wapl*^Δ*/+*^ mice show modest changes in expression levels across hundreds of genes. The genes that are dysregulated in *Wapl*^Δ*/+*^ overlap in large part with genes dysregulated in *Nipbl^+/−^* brain samples. Our results also show that gene expression changes in *Nipbl^+/−^* mice are typically corrected (at least partially) by decreasing *Wapl* dosage. Similarly, expression changes in *Wapl*^Δ*/+*^ mice are corrected by decreasing *Nipbl* dosage. These results are consistent with a model in which cohesin dynamics play an important role in regulating gene expression. Last, we show that decreasing *Wapl* dosage partially rescues *Nipbl*-dependent embryonic growth defects but does not rescue perinatal lethality in *Nipbl^+/−^* pups.

## RESULTS

### *Wapl* loss of function is preimplantation lethal

**Fig. 1.**
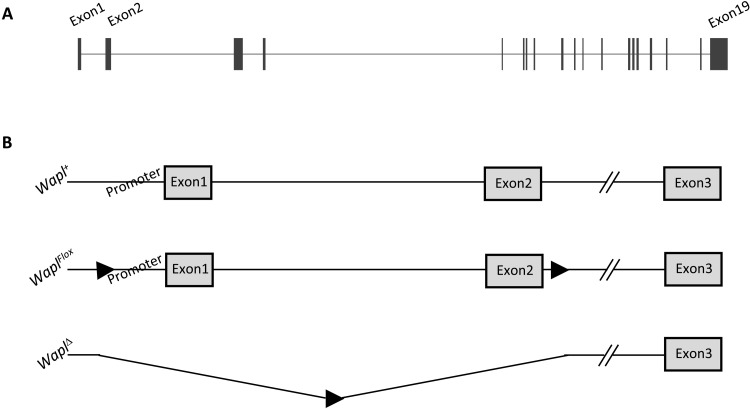
Cartoon depiction of wild-type and mutant Wapl alleles. (**A**) The *Wapl* gene is encoded on 74,056 bp on mouse chromosome 15. Here, we depict the 19 exons of the predominant isoform. For all known isoforms, translation initiates in exon 2. (**B**) Wild-type and mutant alleles used in this study. *Wapl^Flox^* carries *loxP* insertions at −517 and +3641 bp (all numbers are relative to the major transcriptional start site). Thus, cre-mediated recombination results in deletion of the *Wapl* promoter and exons 1 and 2 to form the *Wapl*^Δ^ allele. *LoxP* site, filled arrowhead.

Wild-type and mutant alleles of *Wapl* are depicted in Fig. 1. Mice carrying a conditional *Wapl* allele (*Wapl^Flox^*) were generated as described in Materials and Methods. In brief, *loxP* sites were inserted upstream of the *Wapl* promoter and downstream of exon 2. Both homozygous and heterozygous mice carrying this allele are viable and fertile. To generate *Wapl*^∆^ mice, we crossed *Wapl^Flox/+^* males with females homozygous for the *E2a-Cre* transgene (JAX #003724) and then backcrossed progeny to C57BL/6J (JAX #000664). The *Wapl*^∆^ allele was expected to be a null allele based on the deletion of the *Wapl* promoter, the translation initiation site, and the peptide coding sequences in exon 2. *Wapl*^∆*/+*^ mice were recovered at normal Mendelian frequencies (table S1). However, as expected both from the essential role of WAPL in chromosome segregation and from previous analyses (*34*), *Wapl*^∆/∆^ animals were not recovered at weaning (table S2) or even at the blastocyst stage (table S3). This shows that *Wapl* function is required for early development in mice.

### Decreasing *Wapl* dosage prevents normal mouse development

Although *Wapl*^Δ*/+*^ and *Wapl^Flox/Flox^* mice are each viable and fertile, we could not generate *Wapl*^Δ*/Flox*^ weanlings. This was tested using two different mating schemes. When *Wapl*^Δ*/+*^ and *Wapl^Flox/+^* mice were intercrossed, no *Wapl*^Δ*/Flox*^ weanlings were identified in 30 progeny (table S4). Similarly, when *Wapl*^Δ*/+*^ and *Wapl^Flox/Flox^* mice were intercrossed, no *Wapl*^Δ*/Flox*^ weanlings were identified in 20 progeny (Table 1). However, *Wapl*^Δ*/Flox*^ pups are present in expected Mendelian frequencies at embryonic day 17.5 (E17.5; Table 1). This *Wapl*^Δ*/Flox*^ phenotype is reminiscent of the effect of reducing *Nipbl* gene dosage: *Nipbl^+/−^* heterozygotes are present as late-stage embryos but die before weaning. Together, these data show that the *Wapl^Flox^* allele is not completely wild type and that decreasing *Wapl* gene dosage is detrimental to mouse development.

**Table 1.** *Wapl*^Δ*/Flox*^ weanlings are not viable. χ^2^ = 20.000 with 1 df; *P* < 0.001. But *Wapl*^Δ*/Flox*^ E17.5 embryos are found at the expected Mendelian frequency. χ^2^ = 0.077 with 1 df; *P* = 0.7815.

**Cross: *Wapl*^Δ*/+*^ x *Wapl*^*Flox/Flox*^**					
**Collect animals at postnatal day 21**			**Collect animals at embryonic day 17.5**		
Genotype	*Wapl* ^Δ*/Flox*^	*Wapl^+/Flox^*	Genotype	*Wapl* ^Δ*/Flox*^	*Wapl^+/Flox^*
Observed	0	20	Observed	7	6
Expected	10	10	Expected	6.5	6.5

### Genetic interactions between *Wapl* and *Nipbl* can be queried by generating double heterozgyotes

**Fig. 2.**
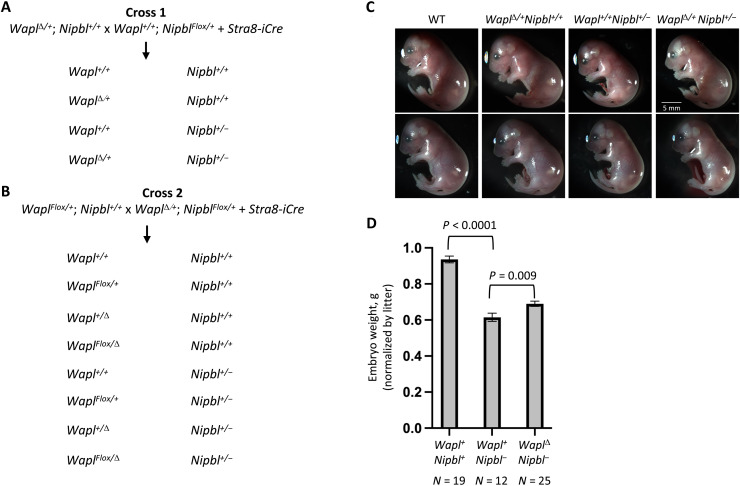
Generating E17.5 *Wapl^Δ/+^*; *Nipbl*^*+/−*^ double heterozygotes along with wild-type littermates and littermates that are heterozygous for loss-of-function mutations in either *Wapl* or *Nipbl*. (**A** and **B**) Crosses used to generate animals for this study. *Nipbl^+/−^* mice die before weaning. Therefore, *Nipbl* mutants are maintained as *Nipbl^Flox/+^* animals. The *Stra8-icre* transgene expresses Cre recombinase in premeiotic male germ cells. Thus, *Nipbl^Flox/+^ Tg(Stra8-icre)* sires generate either *Nipbl^−^* or *Nipbl^+^* sperm. Because the *Stra8-icre* transgene is expressed only in premeiotic germ cells and not in any other tissues, the maternally inherited *Wapl^Flox^* allele in cross 2 is not recombined in the progeny animals. (**C**) Images of cross 1 (top row) and cross 2 (bottom row). (**D**) *Nipbl* mutants show a growth deficiency that is partially compensated by reducing *Wapl* gene dosage. To account for the significant litter-to-litter variations in embryo size, we used a linear regression model that considered both genotype and litter as independent variables. In the analyses depicted here, *Wapl*^Δ*/+*^ and *Wapl*^*Flox/*Δ^ animals were pooled, and we see 24% rescue of the growth defect (*N* = 56; *P* = 0.009). When we excluded animals carrying a *Wapl^Flox^* allele, we see a 19% rescue (*N* = 41, *P* = 0.03).

In *Drosophila*, a developmental defect caused by a *Wapl* dominant-negative allele could be corrected by decreasing *Nipbl* gene dosage (*33*). This led us to hypothesize that decreasing *Wapl* levels in mice might correct developmental defects present in *Nipbl* mutants. To test this, we performed two independent crosses as described in Fig. 2 (A and B). In cross 1, we generated *Nipbl^+/−^* animals in both *Wapl^+/+^* and *Wapl*^Δ*/+*^ backgrounds (Fig. 2A). In cross 2, we generated *Nipbl^+/−^* mice in *Wapl^+/+^*, *Wapl^Flox/+^*, *Wapl*^Δ*/+*^, and *Wapl*^Δ*/Flox*^ backgrounds (Fig. 2B). We assayed survival to weaning, embryonic growth, and brain transcriptomes to test whether decreased *Wapl* gene function would ameliorate *Nipbl^+/−^* phenotypes.

### Reduced *Wapl* function does not rescue *Nipbl^+/−^* postnatal lethality

We analyzed 79 progeny from cross 1, genotyping weanlings at postnatal day 21 (Table 2). *Wapl* inheritance followed normal Mendelian patterns: 38 mice were *Wapl*^Δ*/+*^, and 41 mice were *Wapl^+/+^* (χ^2^ = 0.114, 1 df; *P* = 0.74). However, no *Nipbl^+/−^* animals were identified. We next analyzed 58 progeny from cross 2 (Table 3). Again, *Wapl* inheritance followed the expected patterns, but no *Nipbl^+/−^* animals were identified. Thus, neither the *Wapl*^Δ*/+*^ nor the *Wapl*^Δ*/Flox*^ backgrounds facilitated the survival of *Nipbl^+/−^* pups. Similarly, a *Nipbl^+/−^* background did not permit the survival of *Wapl*^Δ*/Flox*^ pups.

**Table 2.** Decreasing *Wapl* dosage does not rescue lethality in *Nipbl^+/−^* mice. Genotypes of 79 weanlings were determined. Expected^1^, numbers of progeny expected assuming that all genotypes survive; χ^2^ = 79.228 with 3 df; *P* < 0.0001. Expected^2^, numbers of progeny expected assuming that *Wapl* haploinsufficiency fully rescues lethality in *Nipbl^+/−^* mice; χ^2^ = 39.671 with 2 df, *P* < 0.0001. Expected^3^, numbers of progeny assuming that *Wapl* haploinsufficiency cannot rescue lethality in *Nipbl^+/−^* mice; χ^2^ = 0.114 with 1 df, *P* = 0.7357.

**Cross: *Wapl*^Δ*/+*^; *Nipbl*^+/+^ x *Wapl*^+/+^; *Nipbl*^*Flox/+*^ *Tg(Stra8-icre)***				
**Genotype**	***Wapl* ^Δ*/+*^ *Nipbl* ^*+/+*^**	***Wapl* ^*+/+*^ *Nipbl* ^*+/+*^**	***Wapl* ^Δ*/+*^ *Nipbl* ^*+/−*^**	***Wapl* ^*+/+*^ *Nipbl* ^*+/−*^**
Observed	38	41	0	0
Expected^1^	19.75	19.75	19.75	19.75
Expected^2^	26.33	26.33	26.33	0
Expected^3^	39.5	39.5	0	0

**Table 3.** Decreasing *Wapl* gene dosage does not rescue lethality in *Nipbl^+/−^* mice, and decreasing *Nipbl* gene dosage does not rescue lethality in *Wapl*^Δ*/Flox*^ mice. Genotypes of 58 weanlings were determined. Expected^1^, numbers of progeny expected assuming that all genotypes survive; χ^2^ = 79.228 with 3 df, *P* < 0.0001. Expected^2^, numbers of progeny expected assuming that *Wapl* haploinsufficiency fully rescues lethality in *Nipbl^+/−^* mice; χ^2^ = 39.671 with 2 df, *P* < 0.0001. Expected^3^, numbers of progeny assuming that *Wapl* haploinsufficiency cannot rescue lethality in *Nipbl^+/−^* mice; χ^2^ = 0.114 with 1 df, *P* = 0.7357.

**Cross: *Wapl*^*Flox/+*^; *Nipbl*^*+/+*^ x *Wapl*^Δ*/+*^; *Nipbl*^*Flox/+*^ *Tg(Stra8-icre)***								
**Genotypes**	***Wapl* ^*Flox/*Δ^ *Nipbl* ^*+/+*^**	***Wapl* ^*+/*Δ^ *Nipbl* ^*+/+*^**	***Wapl* ^*Flox/+*^ *Nipbl* ^*+/+*^**	***Wapl* ^*Flox/+*^ *Nipbl* ^*+/+*^**	***Wapl* ^*Flox/*Δ^ *Nipbl* ^*+/−*^**	***Wapl* ^*+/*Δ^ *Nipbl* ^*+/−*^**	***Wapl* ^*+/*Δ^ *Nipbl* ^*+/−*^**	***Wapl* ^*+/*Δ^ *Nipbl* ^*+/−*^**
Observed	0	19	20	19	0	0	0	0
Expected^1^	7.25	7.25	7.25	7.25	7.25	7.25	7.25	7.25
Expected^2^	0	11.6	11.6	11.6	11.6	11.6	0	0
Expected^3^	0	19.33	19.33	19.33	0	0	0	0

### Reduced *Wapl* function partially rescues *Nipbl^+/−^* embryonic growth deficiency

Previous analyses showed that *Nipbl^+/−^* embryos display a variety of developmental defects whose penetrance varies greatly from animal to animal (*21*, *30*). One phenotype that is consistently observed in *Nipbl^+/−^* embryos is significantly reduced growth. Reduced growth is also a characteristic of CdLS patients and of *Drosophila* heterozygous for loss of *Nipbl* (*35*). Therefore, we tested the effect of *Wapl* deficiency on embryo weight in both *Nipbl^+/+^* and *Nipbl^+/−^* backgrounds.

We identified mice in proestrus or estrus and set up matings at 14:00. Mating pairs were separated by 07:30 the next day, and embryos were collected between 11:00 and 13:00 on E17.5. With cross 1, we generated six litters and 34 embryos. With cross 2, we generated nine litters and 62 embryos. See Fig. 2C for images of representative embryos. Development of wild-type and mutant mice was indistinguishable on the basis of Theiler staging. Wild-type and mutant embryo weights from these two crosses were collected, and data from the two crosses were pooled for evaluation (table S5).

To account for the significant litter-to-litter variations in embryo size (*36*), we analyzed the data by linear regression using a two-factor model that considered both genotype and litter as independent variables. As previously reported, *Nipbl* deficiency leads to reduced embryo size (*Nipbl^+/+^* = 0.936 g, *Nipbl^+/−^* = 0.615 g or 34% decrease; *P* = 2.95 × 10^−13^; Fig. 2D). In contrast, *Wapl* deficiency has a modest effect (4.5% decrease) that is not statistically significant (*P* = 0.14).

Our primary interest was in the effect of *Wapl* deficiency in a *Nipbl^+/−^* background. When controlling for the effects of litter, double heterozygotes (*Wapl*^Δ*/+*^ *Nipbl^+/−^* or *Wapl*^Δ*/Flox*^ *Nipbl^+/−^*) are significantly larger than *Nipbl^+/−^* embryos (*Nipbl^+/−^ Wapl*^Δ*/+*^ = 0.690 g, *Nipbl^+/−^ Wapl^+/+^* = 0.615 g or 12% increase; *P* = 0.00932; Fig. 2D). This means that 24% of the *Nipbl^+/−^* growth phenotype was rescued by reducing *Wapl* gene dosage.

### *Wapl* RNA levels are insensitive to *Nipbl* heterozygosity, and *Nipbl* RNA levels are insensitive to *Wapl* heterozygosity

**Fig. 3.**
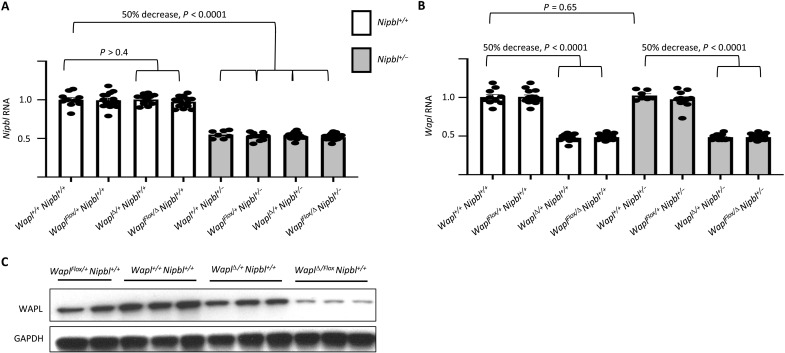
*Wapl* and *Nipbl* transcription and protein expression. (**A** and **B**) RNA analyses. RNAs were isolated from E17.5 brains, analyzed by qRT-PCR, normalized to *GAPDH*, and then normalized to levels in wild-type (*Wapl^+/+^*; *Nipbl^+/+^*) animals. (A) *Nipbl* RNA levels are reduced twofold in *Nipbl^+/−^* heterozygotes but are not affected by mutations in *Wapl*. (B) *Wapl* RNA levels are reduced twofold in *Wapl*^Δ*/+*^ heterozygotes but are not altered by mutations in *Nipbl*. (**C**) WAPL protein analyses. Protein extracts were isolated from E17.5 brains and analyzed by Western blotting. Images were quantitated using NIH ImageJ software and normalized to GAPDH and then to levels seen in wild-type samples. *Wapl^Flox/+^* = 68 ± 18%; *Wapl*^Δ*/+*^ = 64 ± 6% protein levels (*N* = 3, *P* = 0002); *Wapl*^Δ*/Flox*^ = 9 ± 4% (*N* = 3, *P* < 0.001).

We isolated total RNA from E17.5 embryonic brains and quantitated *Wapl* and *Nipbl* expression by quantitative reverse transcription polymerase chain reaction (qRT-PCR). Brains from mice heterozygous for the *Nipbl^−^* allele show 50% loss of *Nipbl* RNA (*P* < 0.001; Fig. 3A). Similarly, brains from mice heterozygous for the *Wapl*^Δ^ allele show 50% reduction in *Wapl* RNA (*P* < 0.001; Fig. 3B).

Several previous studies suggested that *Nipbl* RNA synthesis might be autoregulated since null alleles resulted in *Nipbl* RNA levels that were up to 65% of that seen in wild-type tissues (*21*, *30*, *35*, *37*). However, in this study, *Nipbl* levels in mutant brains are almost exactly 50% of those in wild-type samples. To address this discrepancy more fully, we analyzed RNAs isolated from E17.5 livers. In *Nipbl^+/−^* samples, *Nipbl* RNA was reduced to 48.9% of wild-type levels (*N* = 4, *P* = 0.005), but *Wapl* RNA levels were unaffected (93.5% expression relative to wild type, *P* = 0.26). In *Wapl*^Δ*/+*^ samples, *Wapl* RNA was reduced to 47.5% of wild-type levels (*N* = 4, *P* < 0.001), while *Nipbl* expression was at 90.1% relative to wild type (*P* = 0.35). Thus, in these two developmental stages and in this strain background, we did not see evidence for *Nipbl* (or for *Wapl*) autoregulation at the level of RNA synthesis.

As described above, *Wapl*^Δ*/Flox*^ pups do not survive to weaning, indicating that the *Wapl^Flox^* allele is a *Wapl* hypomorph. However, in Fig. 3A, we saw that the *Wapl^Flox^* allele does not alter *Wapl* RNA levels. We therefore analyzed protein extracts isolated from mutant embryos and saw that WAPL protein levels are significantly reduced by the *Wapl^Flox^* mutation (Fig. 3C). We do not have a good explanation for why the *loxP* insertions affect protein synthesis. However, these Western blot analyses confirm both that *Wapl^Flox^* is a true hypomorph and that it cannot be used as a pseudo–wild-type allele for conditional depletion studies.

For this study, the data in Fig. 3 (A and B) confirm that *Nipbl* levels are not altered by *Wapl* mutations and that *Wapl* RNA levels are not altered by *Nipbl* mutations. This information was essential for designing and interpreting the transcriptome analyses described below.

### Decreased *Wapl* and *Nipbl* each generate broad transcriptome changes in embryonic brains

RNA sequencing (RNA-seq) data were generated from whole-brain tissue derived from female E17.5 embryos. Twenty-two samples were sequenced: five *Wapl^+/+^ Nipbl^+/+^* (referred to as *WT*), seven *Wapl*^∆*/+*^, four *Nipbl^+/−^*, and six *Wapl*^∆/+^ *Nipbl^+/−^* double heterozygotes. After quality control assessment, 1 *Nipbl^+/−^* sample was removed from further analysis because of poor read generation, while the remaining 21 samples were assessed to be of good quality (fig. S1).

**Fig. 4.**
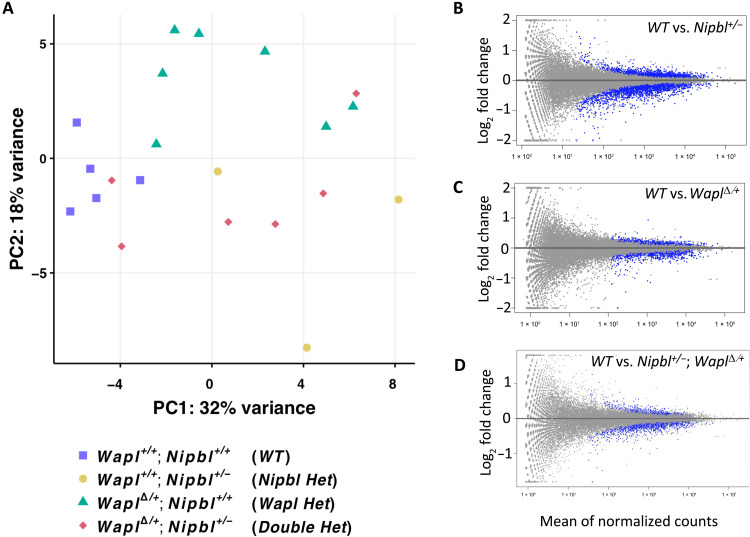
Transcriptome analyses. (**A**) PCA was performed as described in Materials and Methods. Wild-type replicates (purple squares) are tightly clustered. *Nipbl^+/−^* (yellow circles) and *Wapl*^Δ*/+*^ (green triangles) replicates show greater sample-to-sample variation but still cluster into distinct groups. In contrast, double-heterozygote replicates (red diamonds) show high variability and individual samples cluster with both wild-type and individual heterozygotes. Pairwise PCAs comparing wild-type replicates to replicates of each mutant genotype are depicted in fig. S2. A heatmap of sample-to-sample distance is shown in fig. S3. (**B** to **D**) DEGs. MA plots for *wild type* versus *Nipbl^+/−^* (B), *wild type* versus *Wapl*^Δ*/+*^ (C), and *wild type* versus *Nipbl^+/−^*; *Wapl*^Δ*/+*^ samples (D). Blue dots denote genes that are differentially expressed. In (B) to (D), DEGs are normally distributed regarding their expression levels in wild-type samples. Also, for (B) to (D), the change in expression in mutant samples is typically modest: >98% of DEGs show fold changes of less than 2. Volcano plots for these data are presented in fig. S3.

Principal components analysis (PCA) illustrates the segregation of replicates by genotype for *WT*, *Wapl*^Δ*/+*^, and *Nipbl^+/−^* samples (Fig. 4A and fig. S2). However, double-heterozygote replicates do not cluster. Heatmaps of sample-to-sample distances, an independent measure of variance, confirm the variation among the biological replicates of double heterozygotes (fig. S3). Note that two double-heterozygote replicates colocalize with the *WT* samples.

While *Nipbl^+/−^* and *Wapl*^Δ*/+*^ replicates cleanly cluster away from *WT* samples, their replicates are more dispersed, indicating greater intragroup variability. This increased intragroup variability is of note in the context of the phenotypic variability observed in *Nipbl^+/−^* mice, where the penetrance of several abnormal neurological phenotypes is less than 50% (*21*).

Differential expression analysis was performed by DESeq2 using a false discovery rate (FDR)–adjusted *P* value significance threshold of 0.10 (*38*). Comparing *WT* to *Nipbl^+/−^* transcriptomes, 1535 genes were significantly up-regulated, and 1971 genes were down-regulated for a total of 3506 differentially expressed genes (DEGs) (Fig. 4B and table S6). Of these DEGs, 3460 (98.7%) were dysregulated by less than twofold. Comparing *WT* to *Wapl*^Δ*/+*^ transcriptomes, a similar pattern of dysregulation was observed; 697 genes were significantly up-regulated, and 730 genes were significantly down-regulated for a total of 1427 DEGs (Fig. 4C and table S7). Of the 1427 DEGs, 1421 (99.6%) were dysregulated by less than twofold. Together, the transcriptomic dysregulation observed in both *Nipbl^+/−^* and *Wapl*^∆*/+*^ brains is consistent with the broad, low-effect transcriptomic dysregulation reported when studying mutations in cohesin-related genes (*20*, *39*, *40*).

**Fig. 5.**
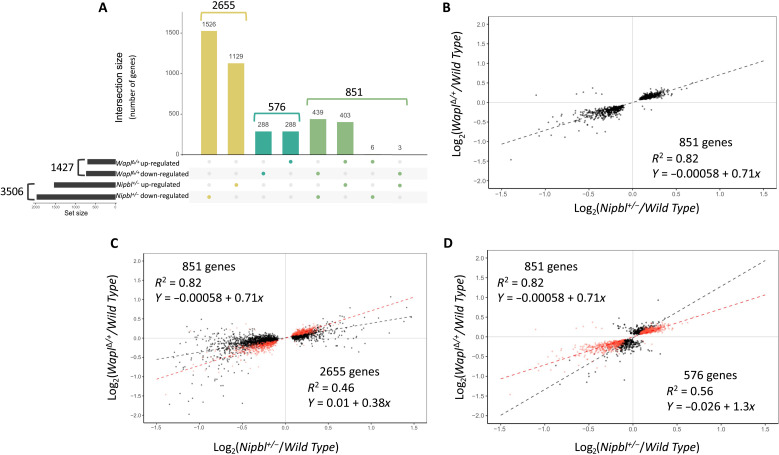
Overlapping transcriptome defects in *Wapl*^Δ/+^ and *Nipbl*^*+/−*^ embryos. (**A**) Intersection of *Nipbl^+/−^* and *Wapl*^∆*/+*^ dysregulated gene sets. A total of 3506 genes are dysregulated in *Nipbl*^+/−^ brains (1535 up-regulated and 1971 down-regulated), and 1427 genes are dysregulated in *Wapl*^∆/+^ brains (697 up-regulated and 730 down-regulated). A total of 2655 genes are dysregulated only in *Nipbl*^+/−^ brains, while 576 genes are dysregulated only in *Wapl*^∆/+^ brains. A total of 851 genes are dysregulated in both *Nipbl*^+/−^ and *Wapl*^∆/+^ brains, the majority of which are dysregulated in the same direction (columns 5 and 6). The overlap is highly significant (*P* < 0.0001, chi square of proportions). (**B**) Linear regression analysis describing dysregulation phenotypes of 851 overlapping DEGs. Each gene (black dot) is plotted according to its dysregulation phenotype in *Nipbl^+/−^* (*x* axis) and *Wapl*^Δ*/+*^ (*y* axis) samples. Note that dysregulation is almost always in the same direction and to a similar degree. Black dashes, line of best fit; correlation coefficient = 0.82. (**C**) Scatterplot describing dysregulation phenotype in all 3506 *Nipbl* DEGs. Red dots show the 851 shared DEGs. Linear regression analyses were performed separately on 851 overlapping DEGs and also on the 2654 genes that were not identified as DEGs in *Wapl* mutants. (**D**) Scatterplot describing the dysregulation phenotype in all 1427 *Wapl* DEGs. Red dots show the 851 shared DEGs. Linear regression analyses were performed separately on 851 overlapping DEGs and also on the 576 genes that were not identified as DEGs in *Nipbl* mutants.

If the transcriptional dysregulation observed in mutant heterozygotes stems from a disruption to cohesin function, one might expect a large set of shared DEGs that define loci where transcription is sensitive to cohesin structures. A total of 851 genes are dysregulated in both *Wapl*^∆/*+*^ and *Nipbl^+/−^* mutants (*P* < 0.0001, chi square of proportions; Fig. 5A). Consistent with previous analyses in *Drosophila* (*41*), dysregulation in *Nipbl* and *Wapl* mutants is almost always in the same direction (Fig. 5B).

The overlap in DEGs is large (25% of *Nipbl* DEGs and 60% of all *Wapl* DEGs), but we suspect that it is still an underestimate of the overlap in transcriptional defects. Linear regression analyses suggest that most *Nipbl* DEGs show a trend toward dysregulation in *Wapl*^Δ*/+*^ samples, even if the *Wapl* effect was not statistically significant enough to score the gene as a *Wapl* DEG (Fig. 5C). Similarly, most *Wapl* DEGs show a trend toward dysregulation in *Nipbl^+/−^* samples (Fig. 5D).

### Decreased *Wapl* dosage partially rescues the transcriptome dysregulation observed in *Nipbl^+/−^* mice

**Fig. 6.**
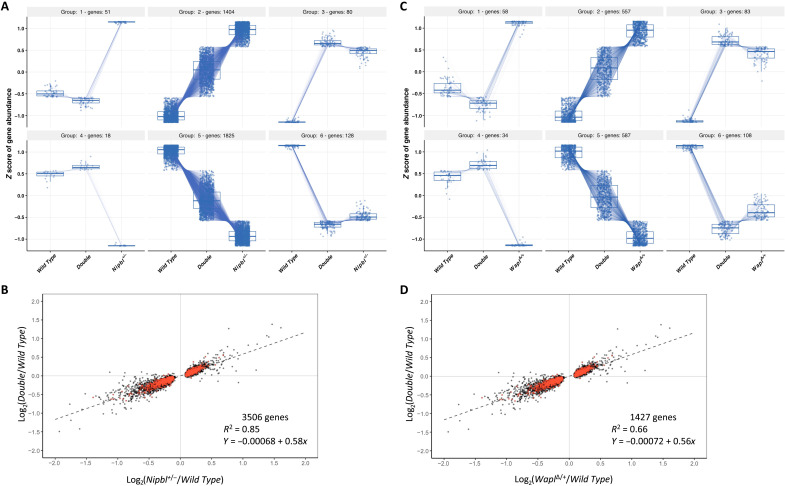
Rescue of transcriptome phenotypes in *Wapl*^Δ*/+*^; *Nipbl*^*+/−*^ double heterozygotes. (**A** and **B**) Rescue of *Nipbl* transcriptome defects. (A) Ninety-four percent of the 3506 genes dysregulated in *Nipbl^+/−^* brains are at least partially rescued by concomitant reduction in *Wapl* gene function. For each gene differentially expressed in *Nipbl* heterozygotes, normalized counts from wild-type, double-heterozygote (*Wapl*^Δ*/+*^; *Nipbl^+/−^*), and *Nipbl^+/−^* samples were analyzed using a DIANA clustering algorithm. (B) Linear regression analyses. For each *Nipbl* DEG, the magnitude of dysregulation in *Nipbl* heterozygotes (*x* axis) is plotted against the magnitude of dysregulation in double heterozygotes (*y* axis). The regression line summarizes the overall effect of *Wapl* mutation. A slope of 1 would indicate no rescue, while a slope of 0 would indicate complete rescue. Here, the slope is 0.58, indicating a 42% rescue. An *R*^2^ value of 0.85 demonstrates the consistency of rescue across the 3506 DEGs. Red dots represent genes that are also significantly dysregulated in *Wapl*^Δ*/+*^ heterozygotes. (**C** and **D**) Rescue of Wapl transcriptome defects. (C) Of the 1427 genes dysregulated in *Wapl*^Δ*/+*^ brains, 87% are at least partially rescued by concomitant reduction in *Nipbl* gene function. For each gene differentially expressed in *Wapl* heterozygotes, normalized counts from wild-type, double-heterozygote (*Wapl*^Δ*/+*^; *Nipbl^+/−^*), and *Wapl*^Δ*/+*^ samples were analyzed using a DIANA clustering algorithm. (D) Linear regression analyses. For each DEG, the magnitude of dysregulation in *Wapl* heterozygotes (*x* axis) is plotted against the magnitude of dysregulation in double heterozygotes (*y* axis). The regression line summarizes the overall effect of *Nipbl* mutation. Red dots represent genes that are also significantly dysregulated in *Wapl^−/+^* heterozygotes.

We focused next on analyses of the double mutant where there are only 1473 DEGs relative to wild-type samples (Fig. 4D). If altered cohesin dynamics are responsible for the transcriptional changes observed in *Wapl*^∆/*+*^ and *Nipbl^+/−^* mutants, it is possible that correcting cohesin dynamics could restore a *WT* transcriptome. This idea led to the hypothesis that a *Wapl*^∆/*+*^ *Nipbl^+/−^* mutant may have a more *WT*-like transcriptome than either a *Wapl*^∆/*+*^ or a *Nipbl^+/−^* mutant, for the double heterozygote would have a *Wapl*:*Nipbl* dosage ratio like *WT*. To test whether the double heterozygote does rescue the transcriptional dysregulation observed in *Nipbl^+/−^* mutants, the 3506 genes differentially expressed between *WT* and *Nipbl^+/−^* were clustered on the basis of their expression levels in *WT*, *Nipbl^+/−^*, and *Wapl*^∆*/+*^ *Nipbl^+/−^* embryonic brains. The unbiased clustering identified six classes of genes, which were annotated as complete rescue of up-regulation (group 1, 51 genes), partial rescue of up-regulation (group 2, 1404 genes), no rescue of up-regulation (group 3, 80 genes), complete rescue of down-regulation (group 4, 18 genes), partial rescue of down-regulation (group 5, 1825 genes), and no rescue of down-regulation (128 genes; Fig. 6A and table S8). In total, 3298 of 3506 (94.1%) *Nipbl* DEGs were identified as exhibiting transcriptional rescue mediated by a reduction of *Wapl* dosage.

Effect ratios were compared between *Nipbl^+/−^* and *Wapl*^∆*/+*^ *Nipbl^+/−^* mutants to further characterize the transcriptional rescue phenotype (Fig. 6B and table S9). Plotting the *Nipbl^+/−^*/*WT* effect ratio on the *x* axis and the *Wapl*^∆*/+*^ *Nipbl^+/−^*/*WT* effect ratio on the *y* axis for each of the 3506 dysregulated genes enabled linear regression to be performed to estimate both the average magnitude of transcriptional rescue and the consistency of transcriptional rescue across the entire dysregulated gene set. Dysregulation in *Wapl*^∆/*+*^* Nipbl^+/−^* mutants was 58% as severe as the dysregulation observed in *Nipbl^+/−^* mutants (see the slope of the regression line in Fig. 6B). This reduction in dysregulation was consistent across the gene set, as demonstrated by a coefficient of determination value (*R*^2^) of 0.85. These results demonstrate the ability of decreased *Wapl* dosage to rescue dysregulation caused by reductions in *Nipbl* dosage.

Similarly, the dysregulation observed in the *Wapl*^∆*/+*^ *Nipbl^+/−^* mutants was less severe than the dysregulation observed in the *Wapl*^∆/*+*^ mutants. Of the 1427 dysregulated genes identified in the *Wapl*^∆/*+*^ embryo, 1236 (86.6%) were clustered into six groups representing transcriptional rescue phenotypes: complete rescue of up-regulation (group 1, 58 genes), partial rescue of up-regulation (group 2, 557 genes), no rescue of up-regulation (group 3, 83 genes), complete rescue of down-regulation (group 4, 34 genes), partial rescue of down-regulation (group 5, 587 genes), and no rescue of down-regulation (group 6, 108 genes; Fig. 6C and table S10). Executing linear regression on plotted effect ratios revealed that dysregulation in *Wapl*^∆/*+*^ *Nipbl^+/−^* mutants was only 56% as severe as the dysregulation in *Wapl*^∆*/+*^ mutants on average (Fig. 6D and table S9). An *R*^2^ value of 0.66 indicates consistency in the magnitude of transcriptional rescue observed.

**Fig. 7.**
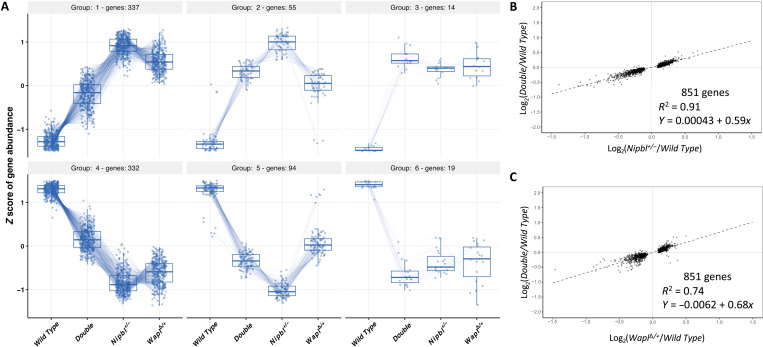
Rescue of overlapping transcriptome defects in *Wapl*^Δ*/+*^; *Nipbl*^*+/−*^ double heterozygotes. (**A**) Of the 851 genes misexpressed in both *Nipbl^+/−^* and *Wapl*^Δ*/+*^ brains, 79% are at least partially rescued by concomitant reduction in either *Nipbl* or *Wapl* gene function. For each gene differentially expressed in both *Nipbl* and *Wapl* heterozygotes, normalized counts from wild-type, double-heterozygote (*Wapl*^Δ*/+*^; *Nipbl^+/−^*), *Nipbl^+/−^*, and *Wapl*^Δ*/+*^ samples were analyzed using a DIANA clustering algorithm. (**B**) Linear regression analyses. For each overlapping DEG, the magnitude of dysregulation in *Nipbl* heterozygotes (*x* axis) is plotted against the magnitude of dysregulation in double heterozygotes (*y* axis). The regression line summarizes the overall effect of *Wapl* mutation. A slope of 1 would indicate no rescue, while a slope of 0 would indicate complete rescue. Here, the slope is 0.59, indicating a 41% rescue. An *R*^2^ value of 0.91 demonstrates the consistency of rescue across the 851 DEGs. (**C**) For each overlapping DEG, the magnitude of dysregulation in *Wapl* heterozygotes (*x* axis) is plotted against the magnitude of dysregulation in double heterozygotes (*y* axis). The regression line summarizes the overall effect of *Nipbl* mutation. Here, the slope is 0.68, indicating a 32% rescue. An *R*^2^ value of 0.74 demonstrates the consistency of rescue across the 851 DEGs.

In Fig. 7, we analyze rescue of the 851 shared DEGs. These results emphasize that transcriptional rescue in the double heterozygotes occurs even when dysregulation in *Nipbl^+/−^* and *Wapl*^∆*/+*^ samples is in the same direction. Forty percent of shared DEGs are up-regulated in both *Nipbl* and *Wapl* heterozygotes and then partially rescued in the double mutant (group 1). Similarly, 39% of shared DEGs are down-regulated in both heterozygotes and then partially rescued in the double mutant (group 4; Fig. 7A and table S11). On average, we see a 41% rescue of *Nipbl* dysregulation (*R*^2^ = 0.91; Fig. 7B) and a 32% rescue of *Wapl* dysregulation (*R*^2^ = 0.74; Fig. 7C).

In Figs. 6 and 7, we analyzed the possible rescue of *Nipbl* and *Wapl* dysregulation phenotypes by comparing the averaged *Wapl*^Δ*/+*^ *Nipbl^+/−^* transcriptome with the averaged *Nipbl^+/−^* and averaged *Wapl*^Δ*/+*^ transcriptomes. However, as discussed above, double heterozygotes show significant sample-to-sample variation (Fig. 4A and figs. S2 and S3). Therefore, we wanted to look at possible rescue in each double-heterozygote sample separately. Heatmaps in fig. S5 show that rescue phenotypes vary among the six samples: Two samples are very similar to wild type, one sample is very similar to the single mutants, and three samples show partial rescue similar in magnitude to the effects described in Figs. 6 and 7.

### Common genetic pathways are disrupted by reduced *Wapl* and by reduced *Nipbl* function

*Nipbl* down-regulated genes are enriched for neuronal-specific functions (table S12). The three most enriched Gene Ontology (GO) classes are synapse organization (125 genes, adjusted *P* = 7 × 10^−40^), regulation of membrane potential (111 genes, adjusted *P* = 4 × 10^−32^), and vesicle-mediated transport in synapse (78 genes, adjusted *P* = 4 × 10^−30^). Other highly enriched pathways are direct downstream consequences of deficient neuronal signaling. Examples include cognition, learning or memory, and locomotory behavior. These dysregulation defects are consistent with and potentially explain the cognitive phenotypes in CdLS.

In contrast, *Nipbl* up-regulated genes (table S13) sort into generic pathways involved in epigenetics (e.g., chromatin organization and histone methylation), protein modifications (e.g., peptidyl-lysine modification), and development (e.g., forebrain development and Wnt signaling). These defects not only can contribute to neurocognitive problems but also are consistent with the broad presentation of phenotypes in CdLS.

*Wapl* down-regulated genes are like *Nipbl* down-regulated genes in that they are enriched in neuron-specific pathways (table S14). The three most enriched GO terms are dendrite development (27 genes, adjusted *P* = 1 × 10^−9^), synapse organization (32 genes, adjusted *P* = 5 × 10^−9^), and cognition (26 genes, adjusted *P* = 3 × 10^−9^). *Wapl* up-regulated genes do not cluster well into specific pathways. We identified only five GO terms with adjusted *P* values of >0.01 (table S15). As with *Nipbl* up-regulated DEGs, these pathways are not neuronal specific. The three most enriched pathways are extracellular matrix organization, collagen metabolic process, and cell signaling. Extracellular matrix and collagen pathways are not enriched among *Nipbl* DEGs.

Since our research goal was to test the ability of reduced *Wapl* function to rescue *Nipbl* defects, we focused on comparing *Nipbl* DEGs that were rescued and those that were not rescued in the *Wapl*^Δ*/+*^ *Nipbl^+/−^* double mutants. Nonrescued genes are tabulated in table S16. Note that rescued and nonrescued sets are similar in total expression and in severity of dysregulation in *Nipbl^+/−^* brains (fig. S4).

Ninety-five percent of *Nipbl* DEGs are at least partially rescued. Consequently, GO term analyses predictably yielded results that are highly similar to those seen when analyzing all Nipbl DEGs. Compare tables S12 and S17 and tables S13 and S18. Analyzing the 208 nonrescued genes identified hormone transport (8 genes, adjusted *P* = 0.004) as the only pathway with multiple genes and a high significance (table S19). Otherwise, we could not identify functional connections among nonrescued *Nipbl* DEGs.

### *Wapl* heterozygosity rescues *Nipbl*-dependent *Protocadherin beta* gene dysregulation

*Protocadherin* genes (*Pcdh*) are organized into three linked subclusters (α, β, and γ) that together span 1 million base pairs (bp) on mouse chromosome 18. Stochastic activation of *Pcdh* genes generates a cell surface identity that allows each neuron to distinguish self from other neurons (*42*, *43*). Since cohesin has been demonstrated to play a critical role in determining the cell-specific expression (*44*), we were interested in examining the role of *Wapl* and *Nipbl* in regulating *Pcdh* gene activity. We saw that each cluster responds differently to disruption of normal *Wapl* and *Nipbl* function.

Consistent with a previous report (*21*), *Nipbl* deficiency results in decreased expression of 21 of 22 genes within the *Pcdh*β cluster to 45 to 72% of wild-type levels with adjusted *P* values ranging from 0.01 to <0.001 (table S20 and fig. S5). Only two genes (*Pcdhb3* and *Pcdhb17*) are dysregulated in *Wapl*^Δ*/+*^ mice, and both are up-regulated. However, all 21 genes dysregulated in *Nipbl^+/−^* brains show rescue in the double mutants (fig. S5C).

*Pcdh*α genes are also uniformly down-regulated in *Nipbl^+/−^* brains (13 of 14 statistically significant with adjusted *P* value of ~0.023 to 0.006). Expression of these genes does not show statistically significant differences in *Wapl^+/−^* samples, but they uniformly show a 6% lower expression as compared to the *WT* (versus 10% in *Nipbl^+/−^* samples). There is no obvious rescue in double mutants (table S20). At the gamma locus, changes in gene expression are very modest (up 1 to 3% in both *Nipbl^+/−^* and Wapl^Δ/−^ samples; down 3 to 5% in *Nipbl^+/−^* Wapl^Δ/−^ samples) and not statistically significant (table S20).

## DISCUSSION

In this mouse study, we generated two novel alleles of *Wapl* and analyzed the effects of reducing *Wapl* gene dosage in *Nipbl^+/+^* and *Nipbl^+/−^* backgrounds. Mouse development is sensitive to *Wapl* gene dosage. *Wapl*^Δ*/+*^ mice are viable and fertile, but *Wapl*^Δ*/*Δ^ embryos die even before implantation. *Wapl*^Δ*/Flox*^ (like *Nipbl^+/−^*) embryos are viable at E17.5 but die soon after birth and are absent at weaning.

*Wapl*^Δ*/+*^ brains display dysregulation of >1400 genes, but the effect at each locus is typically less than two-fold. This pattern is typical for cohesinopathy patients and for cohesinopathy models in mouse, zebrafish, and *Drosophila* and is the same pattern we saw in our study in *Nipbl^+/−^* brains. Dysregulation in *Wapl*^Δ^ and in *Nipbl^−^* heterozygotes was similar not only in overall pattern but also in the identity of affected genes. Among *Wapl* DEGs, 60% are also identified as *Nipbl* DEGs and are almost always dysregulated in the same direction and to a similar degree. The fact that both *Nipbl* and *Wapl* depletion lead to such similar defects is consistent with models that stress the dynamic nature of cohesin localization on the chromosome (*4*, *41*).

Transcriptome defects in *Nipbl^−^* and *Wapl^−^* samples are not intensified in the double heterozygotes. Rather, the dysregulated phenotypes are partially rescued in double heterozygotes. Ninety-four percent of *Nipbl* DEGs and 87% of *Wapl* DEGs show at least partial rescue in the *Wapl*^Δ*/+*^ *Nipbl^+/−^* brains, and there are no clear examples where dysregulation is exacerbated by the double mutation. This is especially intriguing since dysregulation in the single mutants is almost always in the same direction. Thus, *Wapl*/*Nipbl* interactions are a paradigm where two wrongs do make a right. Our data are consistent with those of previous studies. For example, co-depletion of WAPL and NIBPL rescued cell proliferation in mammalian cells (*4*), and reduced *Nipbl* gene function rescued developmental defects associated with a dominant-negative *Wapl* allele in *Drosophila* (*33*). Recently, Liu *et al.* (*15*) found that depleting *Wapl* in mouse embryonic stem cells caused a decrease in expression in cell type–specific genes; decreasing the cohesin component Rad21 caused similar changes in gene expression. These authors proposed that cohesin turnover regulated cell-specific genes by facilitating enhancer-promoter communication. Similarly, in our study, we found that reducing either *Nipbl* (which would reduce cohesin levels and activity) or *Wapl* caused a decrease in the expression of genes important in neuronal cells, while genes that increased expression were less cell type specific. A recent study in human HCT-116 cells also asked whether reducing WAPL levels could correct defects caused by NIBPL reductions and vice versa (*45*). Similar to what we observed, most gene expression changes in cells depleted for NIPBL or WAPL were corrected in the cells depleted for both proteins. Thus, our in vivo results agree with the model that the correct balance of cohesin loading and unloading activities, rather than the absolute amounts of WAPL and NIPBL, is most critical.

Transcriptomes from the *Nipbl^+/−^*, *Wapl*^Δ*/+*^, and *Nipbl^+/−^ Wapl*^Δ*/+*^ mutants have several features in common. In each mutant, many, many genes are dysregulated, but the fold changes are always modest. Also, the mutant transcriptomes show more sample-to-sample variance than do their wild-type littermates. Sample-to-sample variance was especially apparent in double heterozygotes. Double-heterozygote mice are highly consistent in that they each express almost precisely 50% levels of both *Wapl* and *Nipbl* mRNAs. However, they showed variance in dysregulation of downstream target genes. Thus, some samples were indistinguishable from wild type, while one sample showed minimal rescue of *Nipbl* dysregulation. We do not understand the basis for this variability, but it is an important feature when designing and analyzing genetic interaction studies and also when considering methods to evaluate efficacies for potential therapies targeting transcriptional regulation defects in CdLS patients.

## MATERIALS AND METHODS

### Mice

*Nipbl^Flox/+^* mice were generated as described by Santos *et al.* (*30*). Cre recombination of the *Nipbl^Flox^* allele generates a complete loss-of-function allele, *Nipbl^−^*, referred to as *Nipbl^FIN^* in the Santos study. For *Nipbl* genotyping, we followed the protocol outlined by Santos *et al.* using primers B045, B048, and B050.

The *Wapl^Flox^* allele was generated for this study using a two-step process. In step 1, mouse embryonic stem cells (R1 line, 129SV) were electroporated with linearized plasmid pMVW1. pMVW1 includes a 1.8-kb 5′ homology flank and a 4.1-kb 3′ homology flank to direct insertion of a 1.9-kb *NeoR* cassette (flanked with *Frt* elements) plus a 4.2-kb Bam HI–Xba I fragment that carries *Wapl* sequences −517 to +3641 bp (relative to the main transcriptional start site) that are flanked with *loxP* sequences inserted as direct repeats. The linearized plasmid also included a 2.8-kb *Diphtheria Toxin-A* gene for negative selection. G418-resistant colonies were isolated and screened by using one primer from outside the 5′ flank (5′-ACCCGGTAGAATTGACCTGC) and one primer internal to the *NeoR* cassette (5′-GAGGAGGACAGTCTAGGGCA) to identify a 2067-bp band. Homologous recombination on the 3′ end was confirmed by amplification using one primer from outside the 3′ flank (5′-GATGTTCCTATAAGCCAAGAAGGC) and one primer from inside the 3′ *loxP* site (5′-GCAAAACAACCCCTCACTCC) that was followed by nested PCR (5′-GATGTTCCTATAAGCCAAGAAGGC and 5′-AGGGTGCTAATGAGATGGCTC) to identify a 236-bp band. Targeted clones were injected into C57BL/6J blastocysts, and chimeric founders were bred to C57BL/6J females to establish the *Wapl^Flox^+NeoR* line. In step 2, *Wapl^Flox^+NeoR* heterozygotes were crossed to *ROSA26:FLPe* knock-in transgenic females (JAX #003946) to remove the *NeoR* cassette via Flp recombinase–mediated site-specific recombination and thus generate the *Wapl^Flox^* mouse (Fig. 1). Animals were backcrossed to C57BL/6J at least four times before use in this study.

We generated the *Wapl*^Δ^ allele by crossing *Wapl^Flox^* heterozygotes with *E2a-Cre* transgenic females (JAX #003724) to remove the 4.1-bp fragment that includes the *Wapl* promoter, exon 1, intron 1, exon 2, and the first 94 bp of intron 2. Animals were backcrossed to C57BL/6J at least four more times before use in this study.

Genotypes were determined by PCR analysis of guide DNAs (gDNAs) extracted from ear punch samples. For *Wapl^Flox^* genotyping, we used a two-primer assay (5′-GATGTTCCTATAAGCCAAGAAGGC and 5′-AGGGTGCTAATGAGATGGCTC) that yields bands of 208 and 242 bp, which represent *Wapl^+^* and *Wapl^Flox^* alleles, respectively. For *Wapl*^Δ^ genotyping, we used a three-primer assay (5′-AGGTAGGGGACAGAACTCCG, 5′-AGAGAGCCAACGCAGGTAAA, and 5′-AACGCAAGCCTAGCAACCT) that yields bands of 145 and 335 bp, which represent *Wapl^+^* and *Wapl*^Δ^ alleles, respectively. This three-primer assay can also detect the *Wapl^Flox^* allele (291 bp).

For *Strat8-iCre* genotyping (*46*), we followed the protocol provided by The Jackson Laboratory (JAX #017490). Details for all genotyping assays are available in table S21.

For timed matings, we identified mice in proestrus or estrus by cytological evaluation of vaginal lavage samples (*47*). Mating pairs were set up in the early afternoon and then separated early the next morning. All mouse studies were performed according to National Institutes of Health (NIH) and Public Health Service (PHS) guidelines and only after protocols were approved by the *Eunice Kennedy* *Shriver* National Institutes of Child Health and Human Development Animal Care and Use Committee.

### RNA analyses by qRT-PCR

RNAs were isolated from snap-frozen tissue samples using Tri-Pure (Sigma-Aldrich, 11667165001) and RNeasy Micro Kit (Qiagen, 74004), analyzed using a Thermo Fisher Scientific NANODROP 2000c to evaluate purity and yield, and then stored at −70°C. For qRT-PCR, complementary DNA (cDNA) samples were prepared with and without reverse transcriptase using random hexamer primers (Roche, 04 887 352 001). cDNAs were analyzed using SYBR Green (Roche, 04 887 352 001) on the Roche LightCycler 480 II (45 cycles with annealing at 60°C) using primers for *Wapl* (5′-AGAGAGTGTAACAGTGCATAATCC and 5′-ACTGCTGAATCAGGTCTTCACA), *Nipbl* (5′-CTGATGTGGTTGCAGCATGT and 5′-TGAGTACAAGCTTTCTTCACAGGT), and *Gapdh* (5′-TCAATGAAGGGGTCGTTGAT and 5′-CGTCCCGTAGACAAAATGGT). Assay specificity was demonstrated by melting curve analyses and gel electrophoresis. Statistical significance was evaluated using Student’s *t* test.

### RNA-seq library preparation and sequencing

Total RNA was extracted from snap-frozen, whole-brain tissue of female E17.5 embryos using Tri-Pure isolation reagent (Sigma-Aldrich, 11667165001) and Qiagen RNeasy Micro kit (Qiagen, 74004) with on-column deoxyribonuclease I treatment (Qiagen, 74004). Thermo Fisher Scientific NANODROP 2000c was used to evaluate purity and yield, and RNAs were stored at −70°C. Samples with RNA integrity numbers of >9.0 as determined using an Agilent BioAnalyzer were purified by oligo(dT). Libraries were prepared using an RNA Sample Prep V2 kit (Illumina) and sequenced on an Illumina HiSeq2500 platform. Paired-end reads (101 bp) were trimmed to remove adapters using cutAdapt v2.4 and mapped to the mouse genome mm10 (GRCm38.p6) using STAR v2.5.3a (*48*). The mapped reads were then counted using the featureCounts command of subread v1.64 (*49*). Sequencing and alignment quality was assessed using MultiQC v1.9 (*50*).

### Differential expression analysis

Count normalization and differential expression analyses were performed using the DESeq2 package in R (*38*). Before differential expression testing, genes with fewer than 10 normalized counts summed across all samples were removed. After differential expression testing, genes with an FDR-adjusted *P* value of <0.10 were called as differentially expressed and subjected to further analyses. PCAs considered the 2000 most variable genes and were performed using DESeq2’s plotPCA function using normalized, variance-stabilizing transformed counts. Sample-to-sample distances were calculated using the Euclidean distance formula considering the entire transcriptome and plotted using the Pheatmap R package version 1.0.12 (https://cran.r-project.org/web/packages/pheatmap/). The UpSetR package was used to visualize the overlap between DEG lists (*51*).

### Identification of transcriptional rescue

To identify genotype-dependent patterns of gene expression, significant genes were clustered and visualized using the degPatterns function from the DEGreport R package version 1.32.0 (https://bioconductor.org/packages/release/bioc/html/DEGreport.html, code accessed on 25 August 2022). For each gene, degPatterns first averaged the normalized counts across replicates within each genotype group and then implemented a DIANA clustering algorithm to group genes into clusters based on similar shifts in gene expression across genotypes. For visualization, expression values were *z* score–transformed.

To quantify the magnitude and consistency of transcriptional rescue for all genes called as differentially expressed, log_2_-transformed effect ratios were calculated where *WT* gene expression was the common denominator to (i) *Nipbl^+/−^* gene expression, (ii) *Wapl*^∆*/+*^ gene expression, and (iii) *Wapl*^Δ*/+*^ *Nipbl^+/−^* gene expression. Here, gene expression is equivalent to the normalized counts of a given gene averaged across the replicates of a given genotype. Effect ratios for DEGs were then plotted, comparing the magnitude of dysregulation imposed by the single heterozygotes—log_2_(*Nipbl^+/−^*/*WT*) or log_2_(*Wapl*^Δ*/+*^/*WT*)—to the magnitude of dysregulation imposed by the double heterozygotes—log_2_(*Wapl*^Δ*/+*^ *Nipbl^+/−^*/*WT*). Simple linear regression was then performed to obtain a regression line, the slope of which was used to summarize the difference in dysregulation imposed by the plotted genotypes. Note that by plotting log_2_(*Wapl*^Δ*/+*^ *Nipbl^+/−^*/*WT*) on the *y* axis and either log_2_(*Nipbl^+/−^*/*WT*) or log_2_(*Wapl*^Δ*/+*^/*WT*) on the *x* axis, slopes of <1 signify a lessened dysregulation of gene expression in the double heterozygotes. In addition, *R*^2^ values were obtained from the simple linear regression, providing an estimation of the consistency of the observed pattern. Because the plots in Fig. 6 show effects in the double mutants on genes that had been selected on the basis of their being significantly different from wild type in one or both single mutants, it seemed possible that some of the observed rescue was partly due to statistical regression to the mean or by false positives in the DEG lists. To control for this possibility, we repeated these analyses by adding the 1473 genes differentially expressed in double mutants and saw no significant change in the slopes of the regression lines obtained from those shown in Fig. 6. To investigate the rescue of *Nibpl* and *Wapl* dysregulation in each double-heterozygote sample separately, the normalized counts of all *Nipbl* DEGs (3506) or *Wapl* DEGs (1427) were plotted for each sample using the Pheatmap R package version 1.0.12 (https://cran.r-project.org/web/packages/pheatmap/). For visualization, the normalized counts of each gene were *z* score–normalized across samples.

### Protein analyses

Proteins were extracted from E17.5 mice brains in 1 ml of T-PER tissue protein extraction buffer (Thermo Fisher Scientific, 78510) and 10 μl of protease inhibitor cocktail (Thermo Fisher Scientific, 78437). Samples were homogenized using 1.6-mm zirconium beads (BenchMark D1032-15) and centrifuged at 10,000*g* for 10 min at 4°C. The supernatant was spun for 10 additional minutes at 4°C to remove any insoluble material and stored at −80°C for later use. Protein yields were quantified using Bradford protein assay (Bio-Rad, #5000006).Thirty micrograms of protein was fractionated by electrophoresis on 10% SDS–polyacrylamide gel electrophoresis (PAGE) gels at 100 V for 1.5 hours and then transferred to a 0.2-μm nitrocellulose membrane at 100 V for 1.5 hours. The following antibodies were used: WAPL [1:1000; Cell Signaling Technology (CST), D9J1U], glyceraldehyde-3-phosphate dehydrogenase (GAPDH; 1:2000; CST, #2118S), and anti-rabbit immunoglobulin G (IgG), horseradish peroxidase (HRP)–linked antibody (1:5000; CST, #7074).

## Notes

View/request a protocol for this paper from *Bio-protocol*.
