# Supplementary material for: Decreasing Wapl dosage partially corrects embryonic growth and brain transcriptome phenotypes in Nipbl+/− embryos
Source: Sci Adv. 2022 Nov 30;8(48):eadd4136. doi: 10.1126/sciadv.add4136 (PMC9710879; doi:10.1126/sciadv.add4136)
Supplement: Supplementary file 1 — Figs. S1 to S7 [file sciadv.add4136_sm.pdf]

Science Advances
AAAS

Supplementary Materials for

Decreasing *Wapl* dosage partially corrects embryonic growth and brain transcriptome phenotypes in *Nipbl*$^{+/-}$ embryos

Connor M. Kean *et al.*

Corresponding author: Judith A. Kassis, jkassis@mail.nih.gov; Karl Pfeifer, pfeiferk@mail.nih.gov

Sci. Adv. **8**, eadd4136 (2022)
DOI: 10.1126/sciadv.add4136

The PDF file includes:

Figs. S1 to S7
Legends for tables S1 to S21

Other Supplementary Material for this manuscript includes the following:

Tables S1 to S21

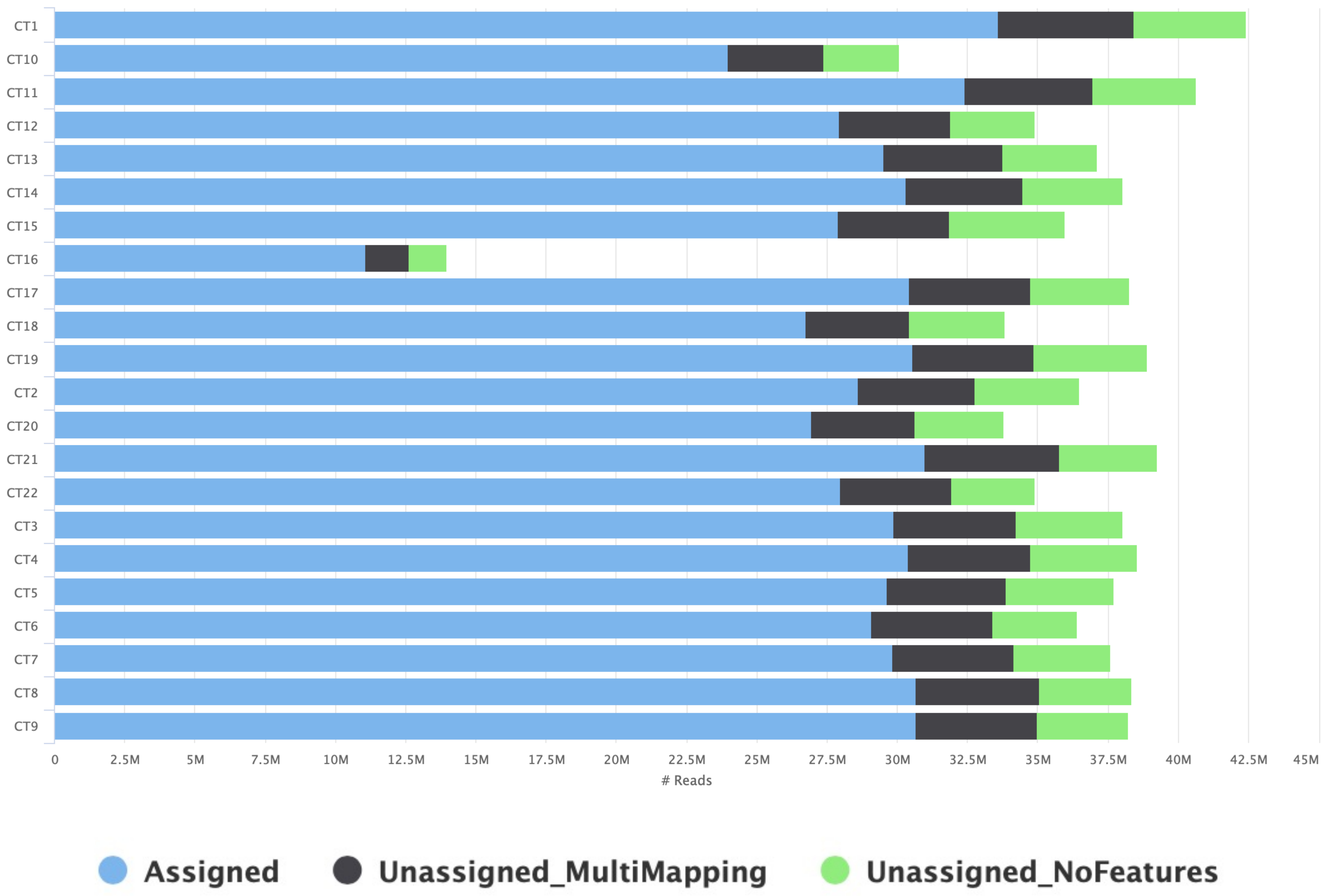

Figure S1. RNA sequencing quality control assessment. RNAs were isolated from E17.5 brains and poly(A) molecules were sequenced as described in Methods. Sequencing quality was analyzed using MultiQC software. This graph depicts the number of uniquely mapped reads for each sample. Sample CT16, from a *Nipbl*$^{+/-}$ animal, was excluded from further analyses based on the failure to generate sufficient reads.

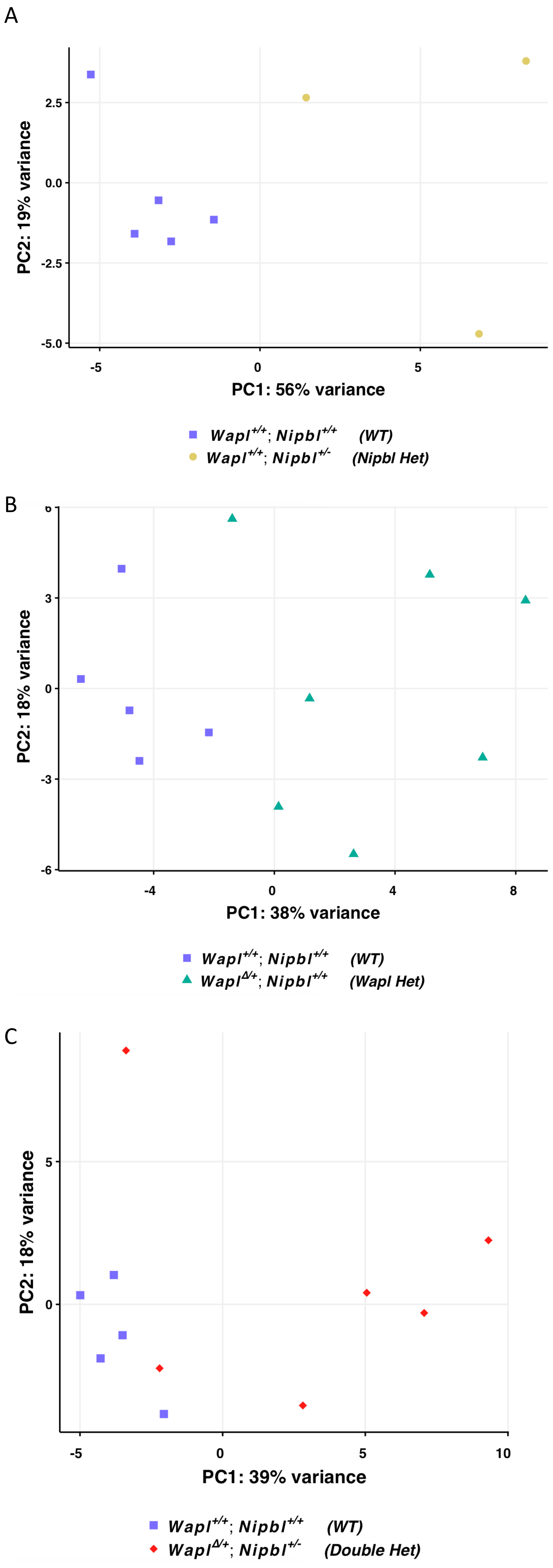

Figure S2. Pairwise Principal Component Analyses. **A-C.** PCAs were performed as described in Methods. Wild type samples (purple squares) are plotted pairwise against **(A)** *Nipbl*$^{+/-}$ (yellow circles), **(B)**, *Wapl*$^{\Delta/+}$ (green triangles), and **(C)** *Wapl*$^{\Delta/+}$; *Nipbl*$^{+/-}$ (red diamonds) replicates. Both *Nipbl*$^{+/-}$ and *Wapl*$^{\Delta/+}$ replicates cluster distinctly from wild type replicates. *Wapl*$^{\Delta/+}$; *Nipbl*$^{+/-}$ double heterozygote replicates do not cluster distinctly from wild type samples; instead, they display varying levels of similarity to wild type samples.

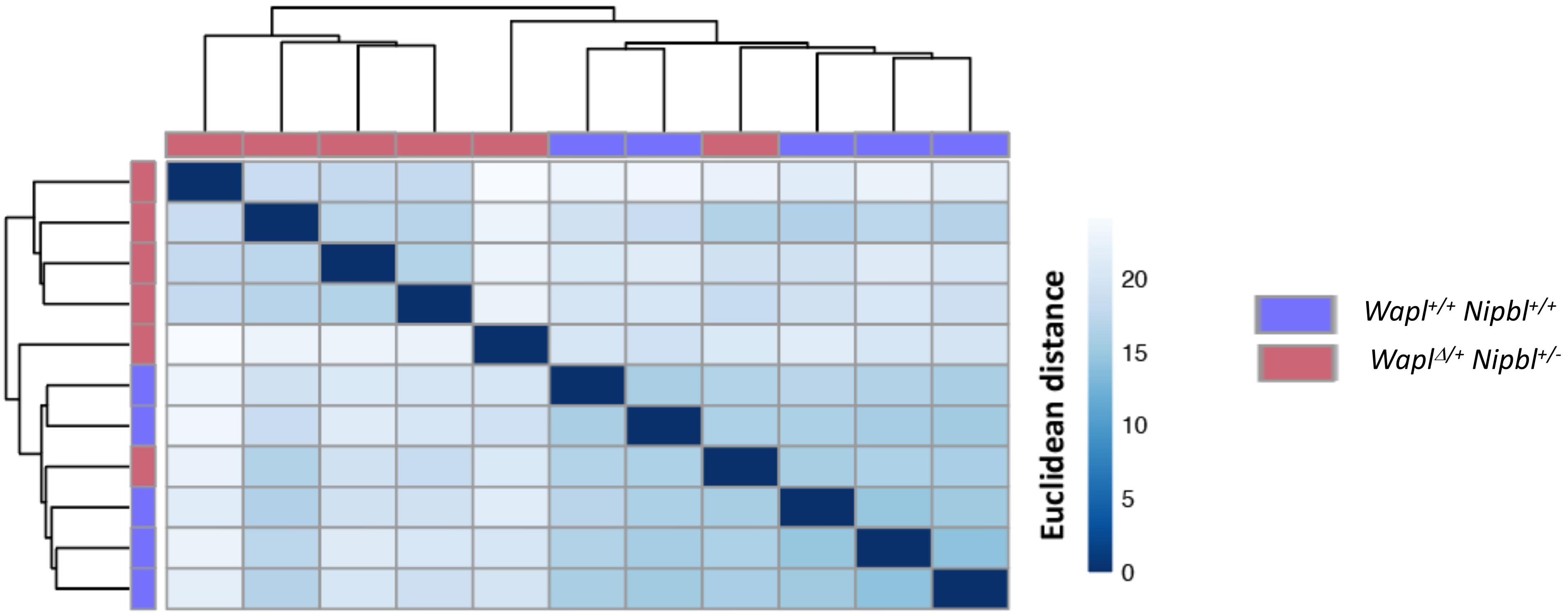

Figure S3. Heatmap of sample to sample distance. Two double heterozygote samples cluster with wild type samples.

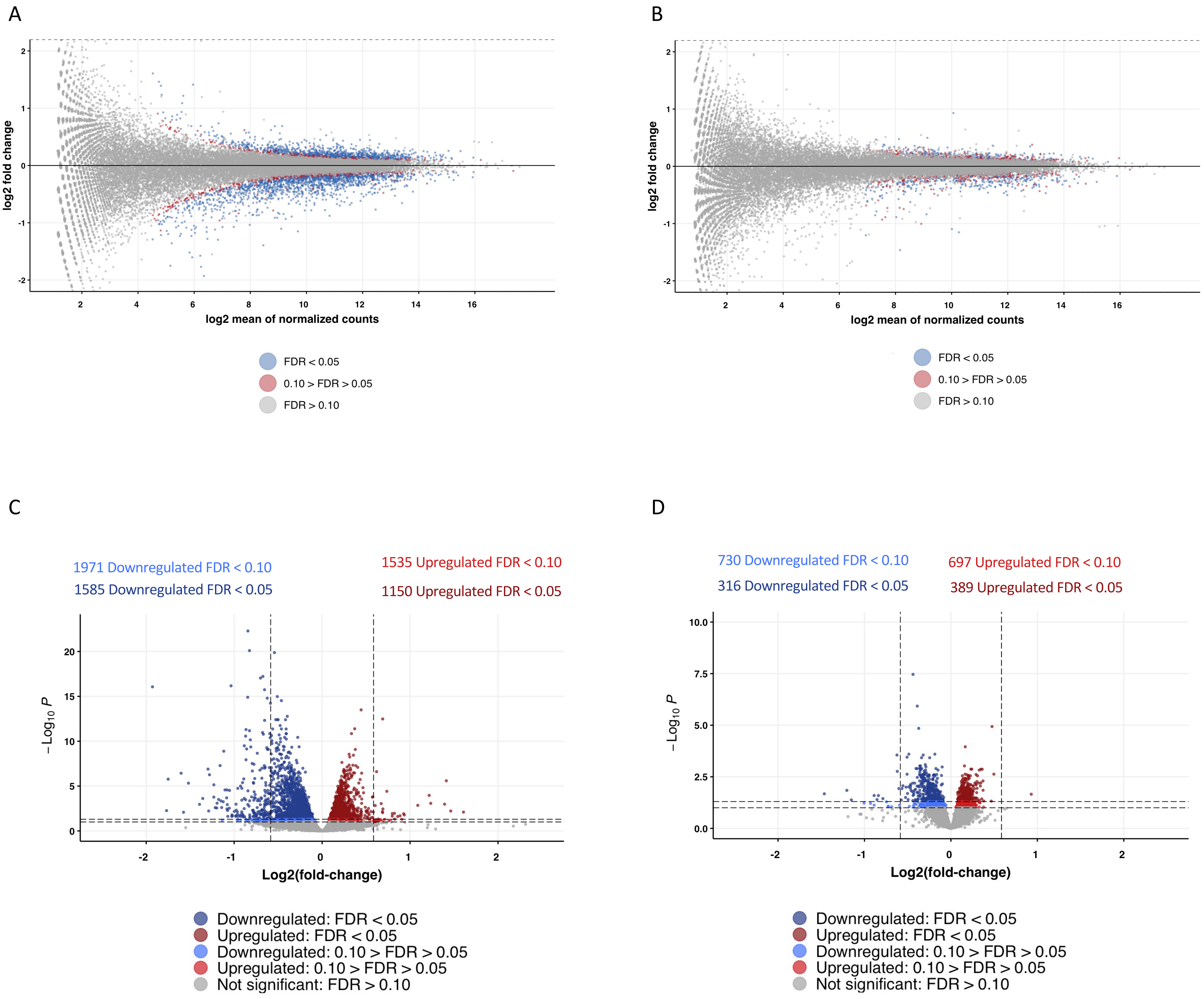

Figure S4. Statistical significance of differentially expressed genes. MA plots for wild type vs. *Nipbl*$^{+/-}$ **(A)** and wild type vs. *Wapl*$^{\Delta/+}$ **(B)** samples. Blue dots denote genes that are called as differentially expressed using an FDR threshold of 0.05. Red dots denote additional genes that are called as differentially expressed when using an FDR threshold of 0.10. Lowering the FDR threshold results in fewer DEG calls across the entire range of expression levels in both comparisons. Volcano plots for wild type vs. *Nipbl*$^{+/-}$ **(C)** and wild type vs. *Wapl*$^{\Delta/+}$ **(D)** samples. Light blue dots (downregulated) and light red dots (upregulated) represent genes that are called as DE using an FDR threshold of 0.10 but are not called as DE using an FDR threshold of 0.05. Dark blue dots and dark red dots represent genes that are called as DE using either FDR threshold. Genes *Nipbl* (adjP = 9.65E-39) and *Pcdhb17* (adjP = 7.71E-37) are not displayed on panel **C** while *Wapl* (adjP = 1.52E-65) is not displayed on panel **D**.

A

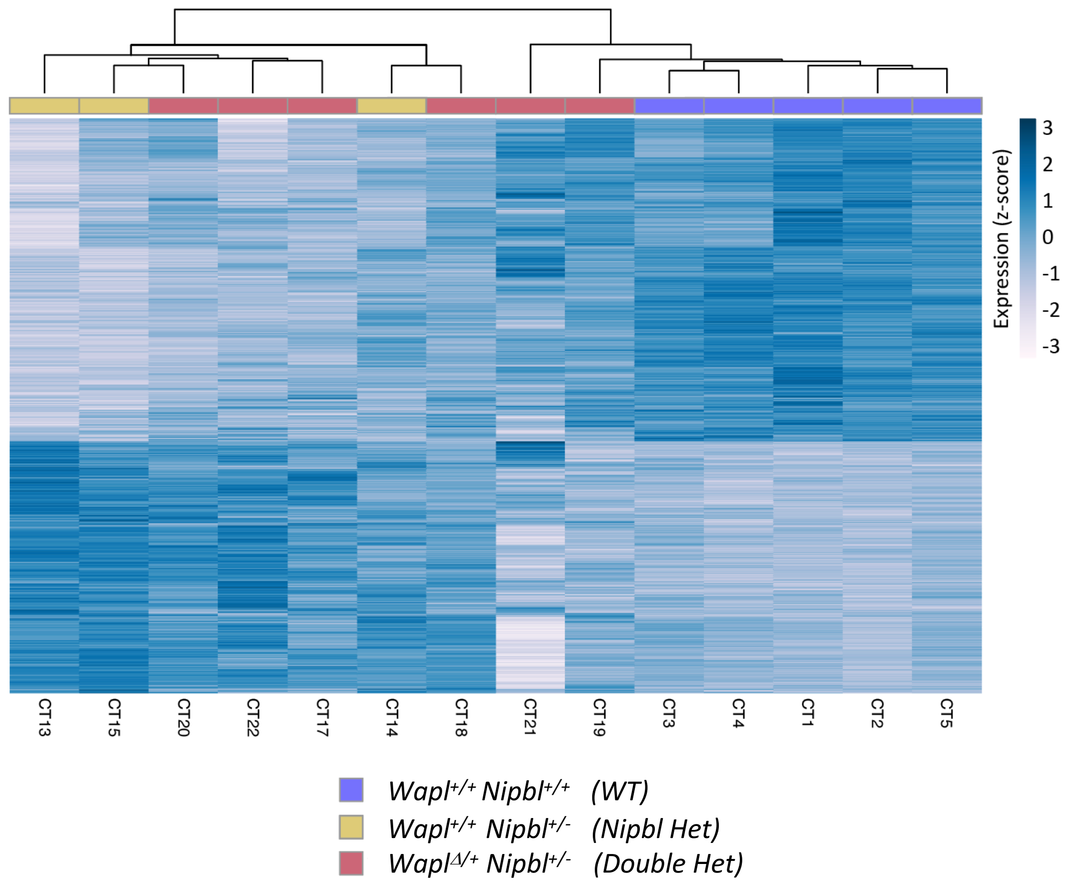

B

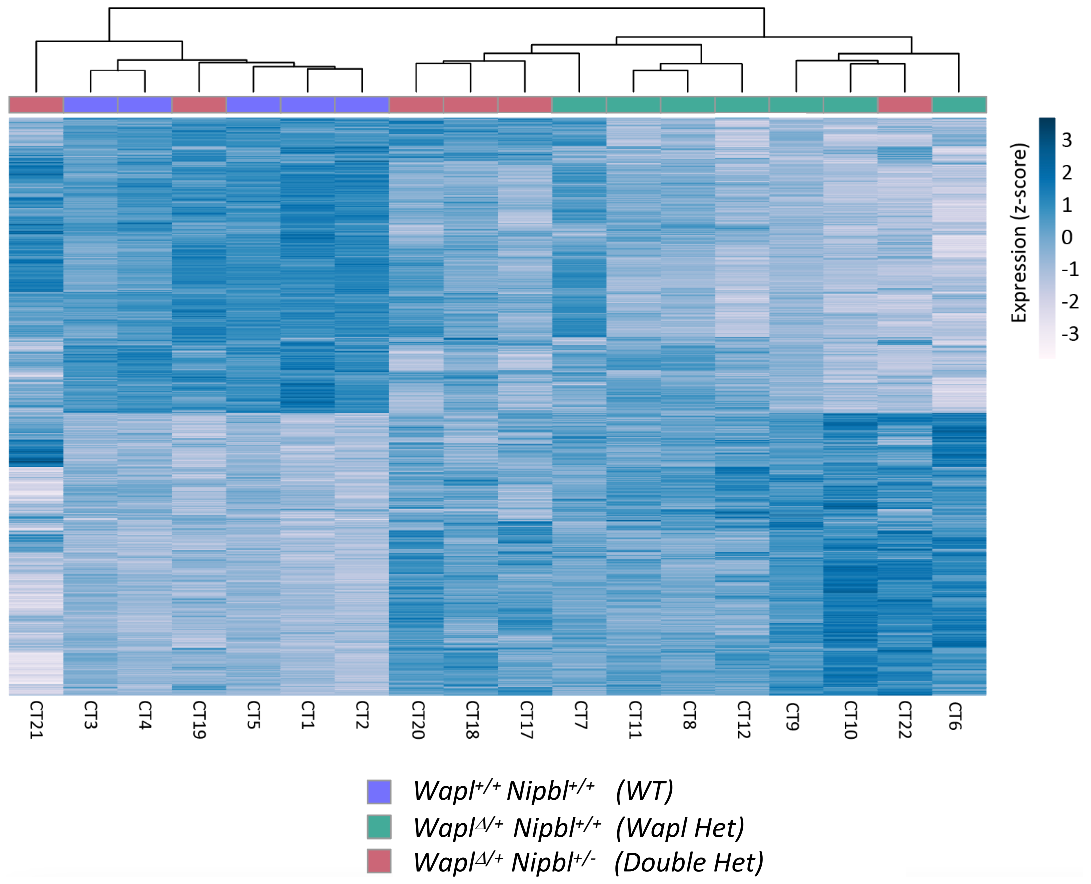

Figure S5. Heatmaps comparing expression of differentially expressed genes in wild type, single mutant, and double mutant samples. **A.** Analysis of the 3506 genes that are differentially expressed in *Nipbl*$^{+/-}$ relative to wild type samples. **B.** Analysis of the 1427 genes that are differentially expressed in *Wapl*$^{\Delta/+}$ relative to wild type.

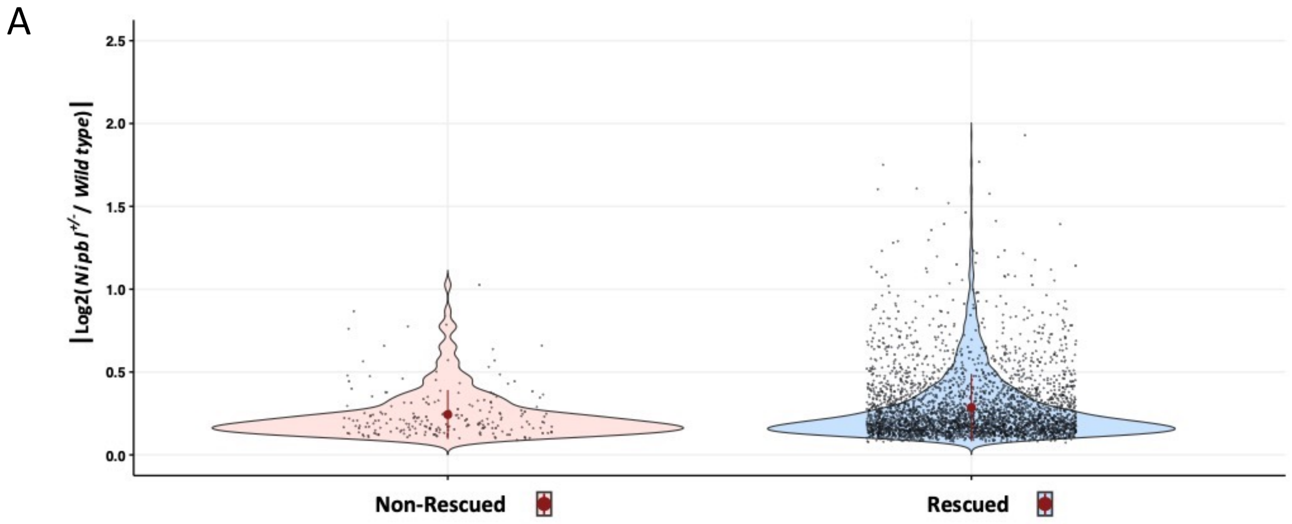

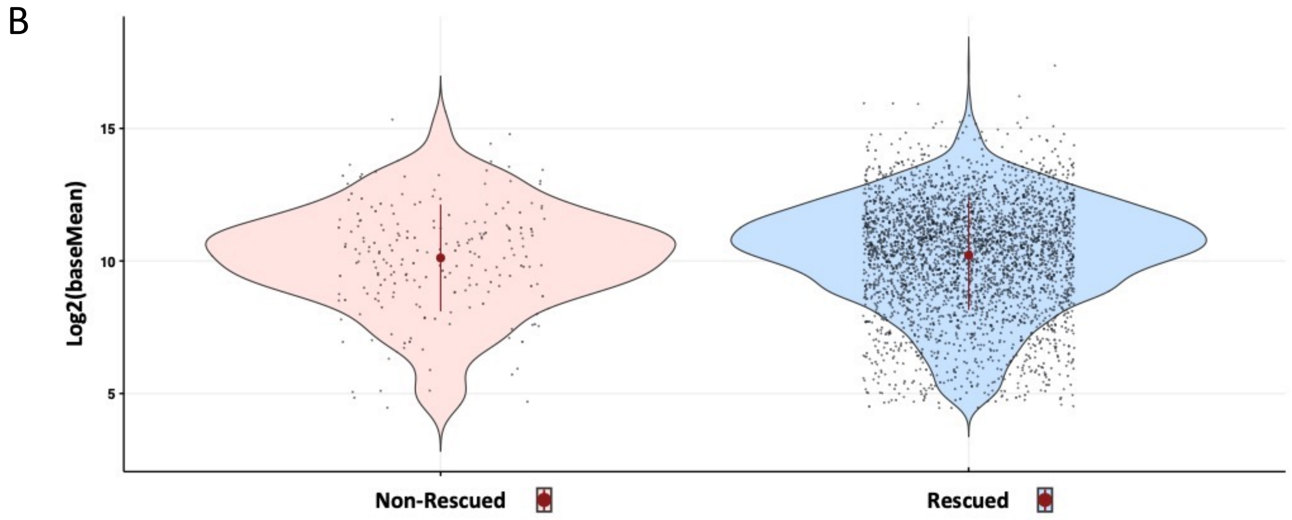

Figure S6. Characterization of genes rescued and not rescued from Nipbl$^{+/-}$ dysregulation. Violin plots displaying the absolute-value log2(Fold-change) of *wild type* vs. *Nipbl$^{+/-}$* brains **(A)** and the level of expression **(B)** of genes that were not rescued by concomitant *Wapl* depletion compared to genes that were rescued by concomitant *Wapl* depletion. Small gray dots represent the values for individual genes, whereas the dark red center dot and line represent the mean value across all genes and the standard deviation across all genes respectively. Non-rescued genes possess a mean log2(fold-change) magnitude of 0.24 compared to a mean of 0.29 for rescued genes. Non rescued genes possess a mean log2(baseMean) value of 10.12 compared to a mean of 10.22 for rescued genes. Rescued genes Gm8210 (log2(FC) = -8.89) and mt-Nd3 (log2(FC) = -3.64) are not displayed on panel **A.**

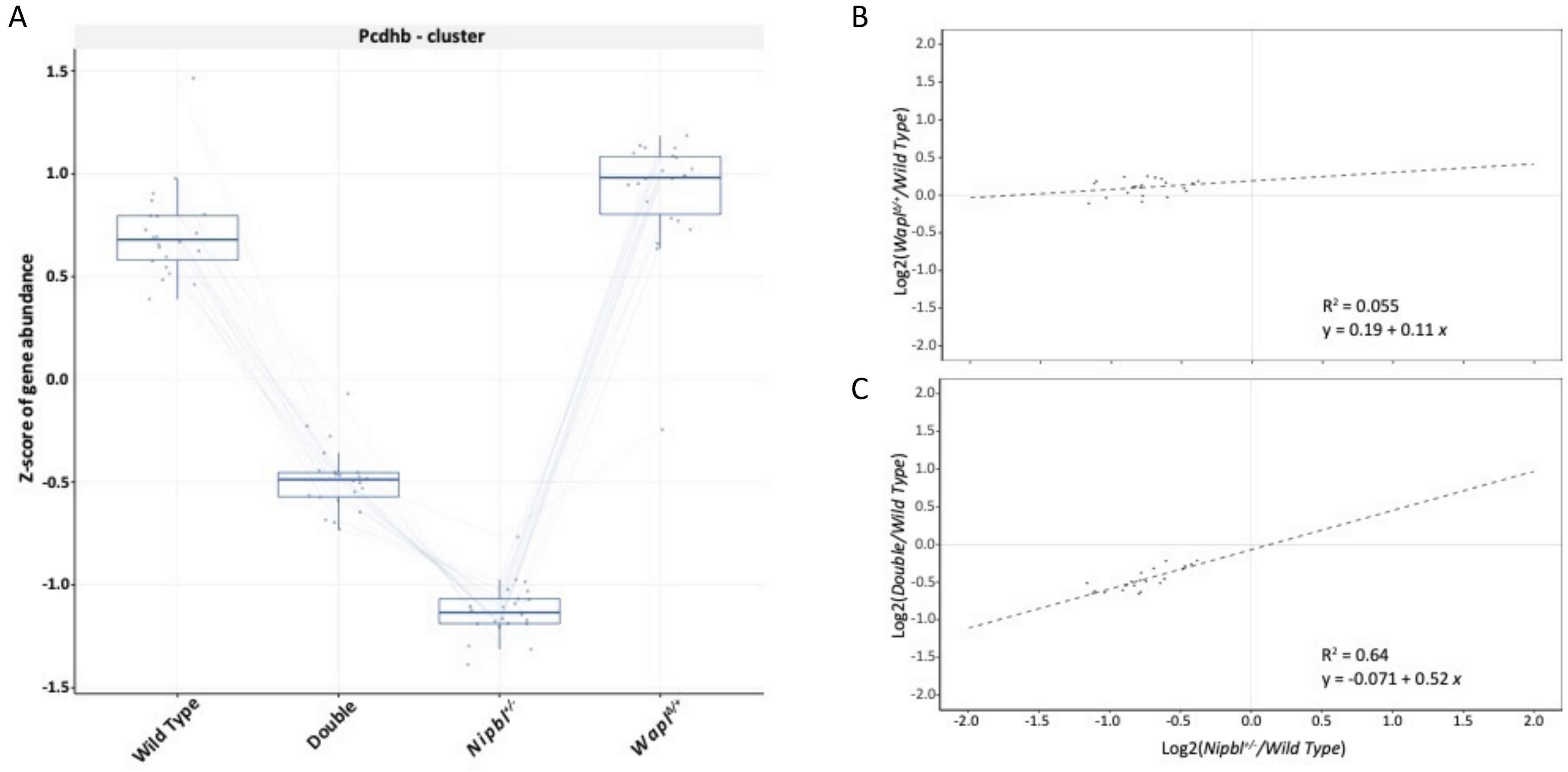

Figure S7. Dysregulation at the *Protocadherin beta* cluster. **A.** 21 *Protocadherinb* genes are dysregulated in $Nipbl^{+/-}$ brains and all are partially rescued by concomitant reduction in *Wapl* gene function. For each beta protocadherin gene, normalized counts from wild type, double heterozygotes ($Wapl^{\Delta/+}$ $Nipbl^{+/-}$), $Nipbl^{+/-}$, and $Wapl^{\Delta/+}$ samples were plotted. **B.** Linear regression analyses describing dysregulation phenotypes of 21 beta protocadherin genes. Each gene is plotted according to its dysregulation phenotype in $Nipbl^{+/-}$ (x axis) and in $Wapl^{\Delta/+}$ (y axes). In $Nipbl^{+/-}$ samples, genes are down regulated to between 45-72% of levels seen in wild type littermates (adjusted p values range between 0.01 to <0.001). In $Wapl^{\Delta/+}$ samples, only two genes are dysregulated (*Pcdhb3* and *Pcdhb17*). These are each upregulated (adjusted p values range between 0.05 and 0.07). **C**. Linear regression analyses describing rescue of *Nipbl*-associated mis-expression phenotypes in $Wapl^{\Delta/+}$ $Nipbl^{+/-}$ double mutants. For each beta cluster gene, the magnitude of dysregulation in double mutants is plotted against the dysregulation in the *Nipbl* heterozygotes. A slope of 1 would indicate no rescue while a slope of 0 would indicate full rescue. Overall, reducing *Wapl* gene dosage rescues about 48% of the *Nipbl* transcriptional defect.

List of Supplementary Tables

Table S1. *Wapl*$^{Δ/+}$ weanings are viable and fertile.

Table S2. *Wapl*$^{Δ/Δ}$ weanings are non-viable.

Table S3. *Wapl*$^{Δ/Δ}$ blastocysts are non-viable.

Table S4. *Wapl*$^{Δ/Flox}$ weanings are not viable.

Table S5. Effect of *Wapl* and *Nipbl* mutations on embryo size at e17.5.

Table S6. Genes differentially expressed in *Nipbl*$^{+/-}$ e17.5 mouse embryonic brain.

Table S7. Genes differentially expressed in *Wapl*$^{Δ/+}$ e17.5 mouse embryonic brain.

Table S8. Genes differentially expressed in *Nipbl*$^{+/-}$ e17.5 mouse embryonic brain grouped by their pattern of expression across *Nipbl* Het, Double Het, and WT genotypes.

Table S9. Effect ratios comparing dysregulation in *Nipbl*$^{+/-}$, *Wapl*$^{Δ/+}$, and double heterozygotes to Wild Type.

Table S10. Genes differentially expressed in *Wapl*$^{Δ/+}$ e17.5 mouse embryonic brain grouped by their pattern of expression across *Wapl* Het, Double Het, and WT genotypes.

Table S11. Genes differentially expressed in both *Nipbl*$^{+/-}$ and *Wapl*$^{Δ/+}$ e17.5 mouse embryonic brain grouped by their pattern of expression across *Nipbl* Het, *Wapl* Het, Double Het, and WT genotypes.

Table S12. Functional Classification of RNAs downregulated in *Nipbl*$^{+/-}$ E17.5 brain samples.

Table S13. Functional Classification of RNAs upregulated in *Nipbl*$^{+/-}$ E17.5 brain samples.

Table S14. Functional Classification of RNAs downregulated in *Wapl*$^{Δ/+}$ E17.5 brain samples.

Table S15. Functional Classification of RNAs upregulated in *Wapl*$^{Δ/+}$ E17.5 brain samples.

Table S16. Differential expression analysis of non-rescued *Nipbl* DEGs.

Table S17. Functional Classification of RNAs downregulated in *Nipbl*$^{+/-}$ E17.5 brain samples but rescued in *Nipbl*$^{+/-}$ *Wapl*$^{Δ/+}$ samples.

Table S18. Functional Classification of RNAs upregulated in *Nipbl*$^{+/-}$ E17.5 brain samples but rescued in *Nipbl*$^{+/-}$ *Wapl*$^{\Delta/+}$ samples.

Table S19. Functional Classification of RNAs dysregulated in Nipbl+/- E17.5 brain samples but not rescued in *Nipbl*$^{+/-}$ *Wapl*$^{\Delta/+}$ samples.

Table S20. Differential expression analysis at the clustered protocadherin locus.

Table S21. PCR primers and conditions.
